# Supplementary figures and images for: Intranasal Administration of Mesenchymal Stem Cell Secretome Reduces Hippocampal Oxidative Stress, Neuroinflammation and Cell Death, Improving the Behavioral Outcome Following Perinatal Asphyxia
Source: Int J Mol Sci. 2020 Oct 21;21(20):7800. doi: 10.3390/ijms21207800 (PMC7589575; doi:10.3390/ijms21207800)

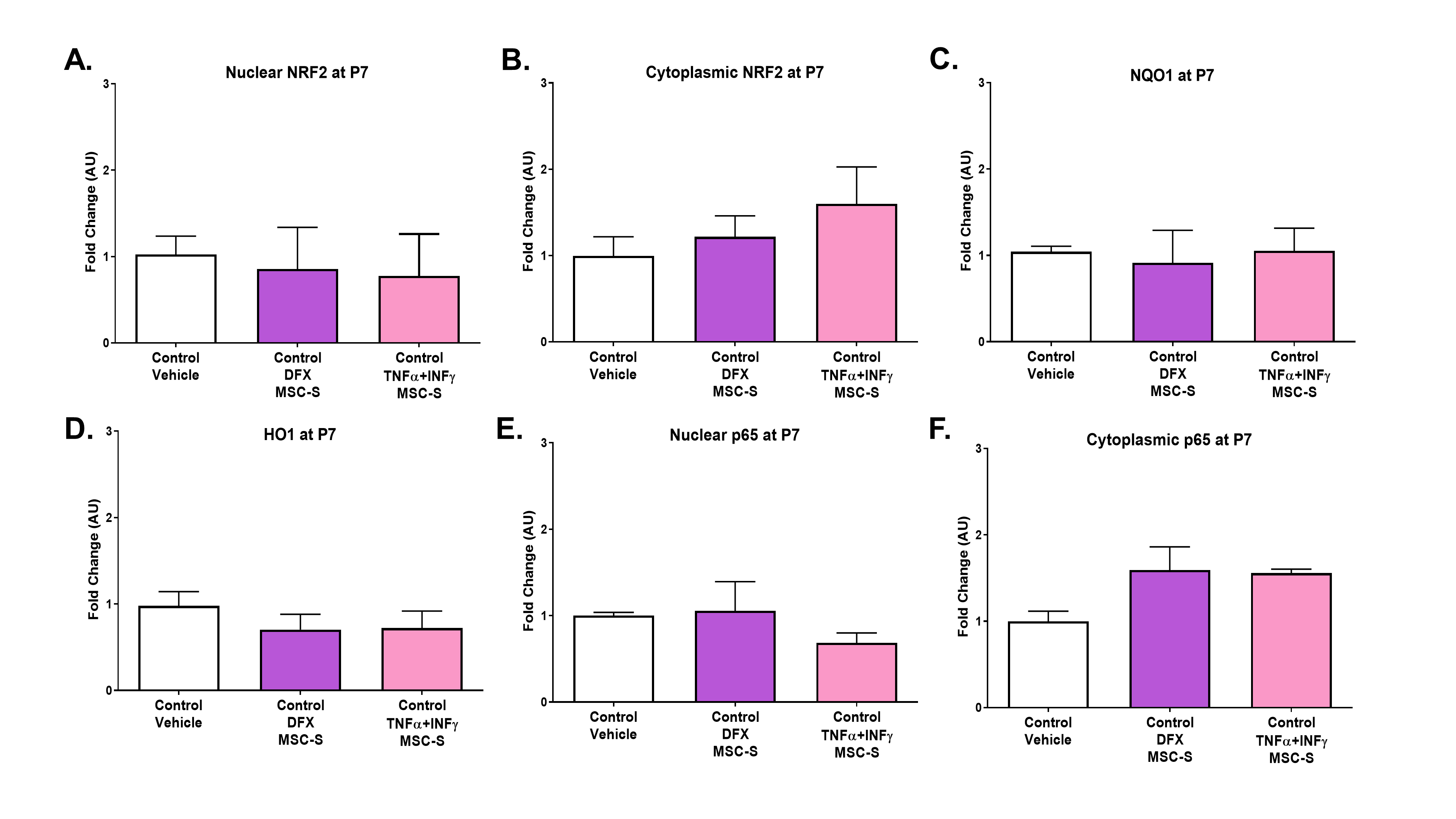

Supplement: Supplementary file 1 [file ijms-21-07800-s001.zip › FarfanetalSupplFiguresIJMS.tiff/FigureS1_ijms.tif]

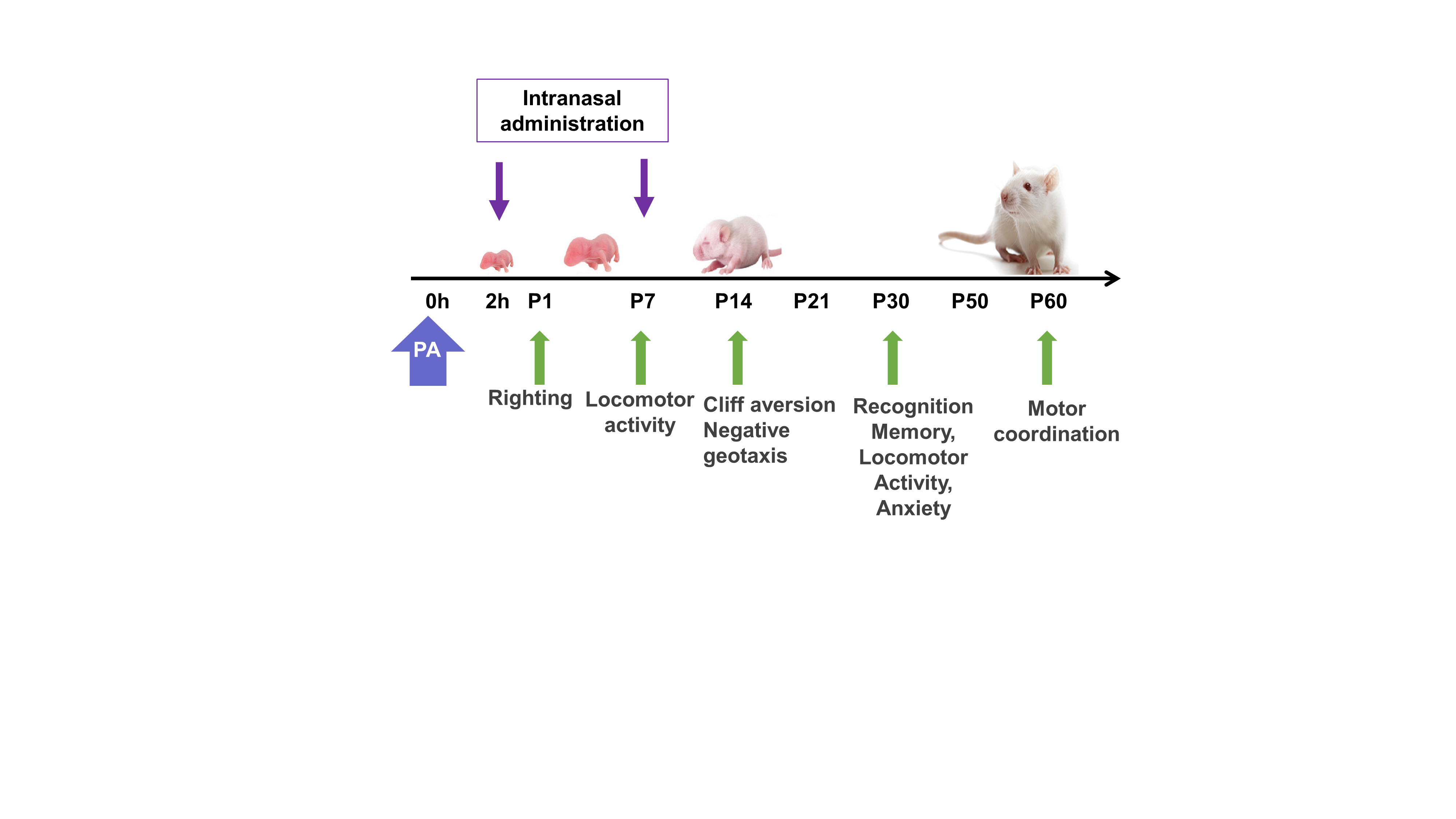

Supplement: Supplementary file 1 [file ijms-21-07800-s001.zip › FarfanetalSupplFiguresIJMS.tiff/FigureS2_ijms.tif]
